# Scanning Probe Microscopy controller with advanced sampling support

**DOI:** 10.1016/j.ohx.2023.e00451

**Published:** 2023-07-07

**Authors:** Miroslav Valtr, Petr Klapetek, Jan Martinek, Ondřej Novotný, Zdeněk Jelínek, Václav Hortvík, David Nečas

**Affiliations:** a Czech Metrology Institute, Okružní 31, 638 00 Brno, Czech Republic; b CEITEC, Brno University of Technology, Purkyňova 123, 612 00 Brno, Czech Republic; c Nenovision, Purkyňova 127, 612 00 Brno, Czech Republic

**Keywords:** Scanning probe microscopy, Adaptive sampling, Field programmable gate array

## Abstract

A low-cost Digital Signal Processor (DSP) unit for advanced Scanning Probe Microscopy measurements is presented. It is based on Red Pitaya board and custom built electronic boards with additional high bit depth AD and DA converters. By providing all the necessary information (position and time) with each data point collected it can be used for any scan path, using either existing libraries for scan path generation or creating adaptive scan paths using Lua scripting interface. The DSP is also capable of performing statistical calculations, that can be used for decision making during scan or for the scan path optimisation on the DSP level.

## Graphical abstract

**Figure:**
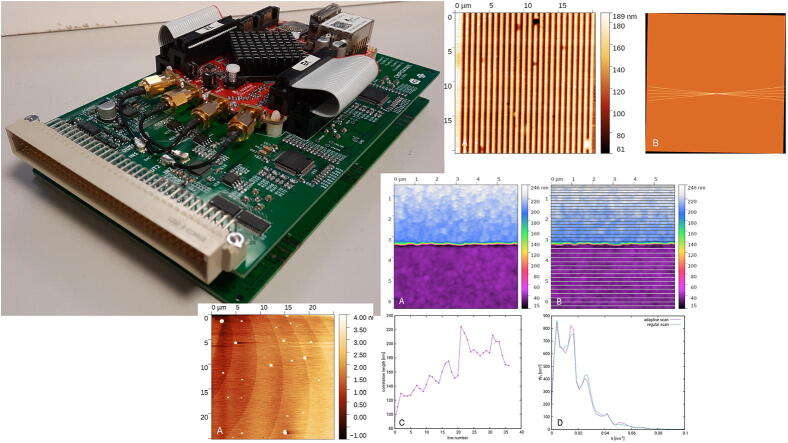

**Specifications table**

**Table:** 

**Hardware name**	Gwyscope
**Subject area**	Engineering and material science
Scanning Probe Microscopy	
**Hardware type**	Imaging tools
Digital Signal Processor	
**Closest commercial analog**	No direct commercial analog is available, some parts of the functionality can be found in independent SPM controllers available on the market, e.g. Nanonis SPM Control System.
**Open source license**	GNU GPL
**Cost of hardware**	1 329 €
**Source file repository**	https://doi.org/10.5281/zenodo.7688987

## 1 Hardware in context

Scanning Probe Microscopy (SPM) [1] is a widely used surface measurement technique, providing maps of surface topography and other physical quantities at nanoscale and microscale. With its many varieties (Atomic Force Microscopy, Magnetic Force Microscopy, Kelvin Probe Force Microscopy, etc.) it can be used in many areas of nanoscience and nanotechnology, as well as in nanoscale metrology. There are numerous producers of SPM equipment, including both whole systems (microscope, controller, data acquisition software) or individual parts (e.g. only controller for customized applications).

A typical SPM measurement produces a rectangular raster pattern representing surface topography, together with additional signals representing the other physical quantities (e.g. contact potential difference) sampled at the same locations as the topography. Such measurement strategy is very simple to set up and the acquired data can be easily visualised and processed, either in built-in SPM manufacturer’s software or in third party commercial or open source software [2], [3], [4]. However, information on the sample is often not distributed evenly and measurement based on a raster pattern can waste time and resources. Therefore, non-raster scanning patterns are being developed [5], [6], [7], including adaptive sampling to measure the important surface features with a higher resolution, or multi-scale sampling to better capture sample statistical properties. This research is usually done with a custom built equipment as the commercial SPM controllers have no or very limited non-raster scanning capabilities. Moreover, most of the SPM data processing software cannot handle such data, making its use even more complicated.

To make the use of non-raster scanning simpler we have worked on different aspects in the past, creating the Gwyscan library for non-raster scan path generation [7] and adding a general XYZ data handling support to an open source software Gwyddion [4]. To demonstrate how the concepts of XYZ data can be used in experimental measurements we have developed the presented hardware. It can use the scan paths generated by Gwyscan library and it collects the data in a way that is compatible with general XYZ data handling in Gwyddion. To create adaptive scanning scripts, it supports scripting via the Lua language [8], [9]. It also supports analysis of local surface statistical properties in the spatial domain (basic statistics, correlation length, period, or mutual shift of two scan lines), which can be used to optimize the sampling pattern to get the best possible ratio between the measurement accuracy and time.

## 2 Hardware description

The hardware is a Digital Signal Processor for SPM, a core component of a SPM electronics that is performing the following operations:

• generating the x,y positions for the scan and providing them to the digital or analog piezo amplifier (which is not part of the presented hardware),
• maintaining feedback loop between probe and sample, e.g. keeping the probe-sample distance constant,
• driving AC signals and performing lock-in detection for advanced SPM regimes,
• communicating with user interface to the microscope (which is not part of the presented hardware) to control the experiment,
• running Lua scripts to perform autonomous scanning.

**Fig. 1:**
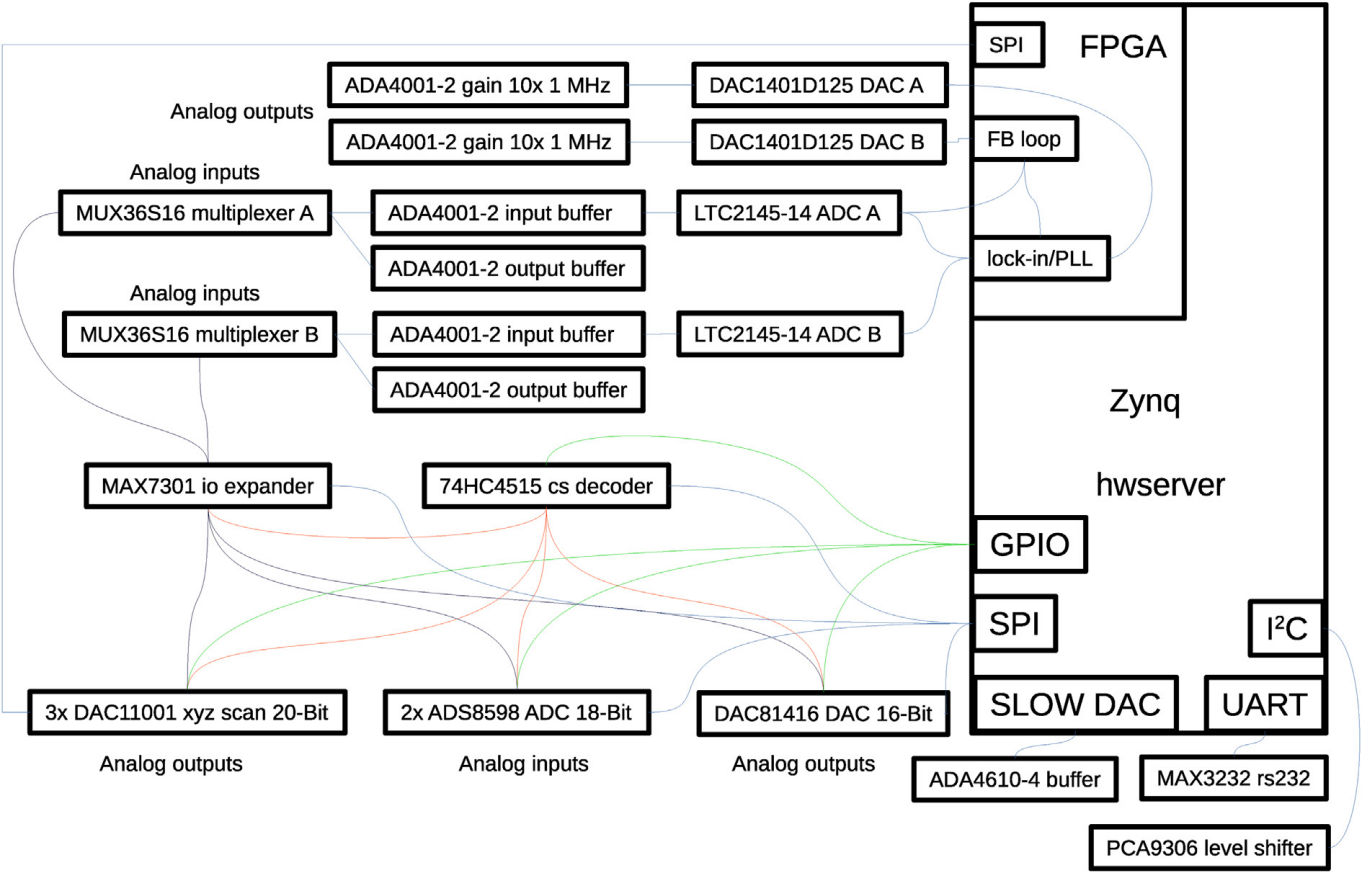
Schematics of the DSP showing all the internal analog and digital interconnections.

**Fig. 2:**
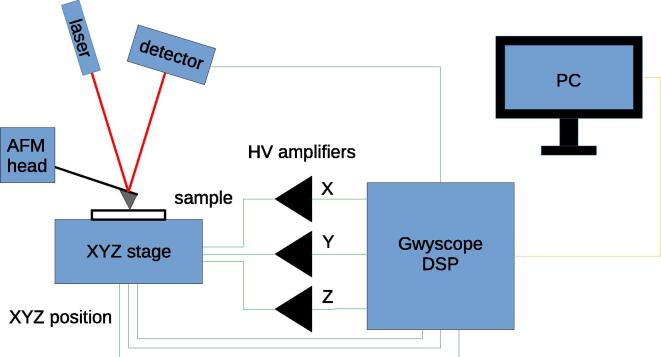
Example showing how the DSP can be used in a SPM hardware. In practice, the scanner configuration and type of used SPM probe can vary.

**Fig. 3:**
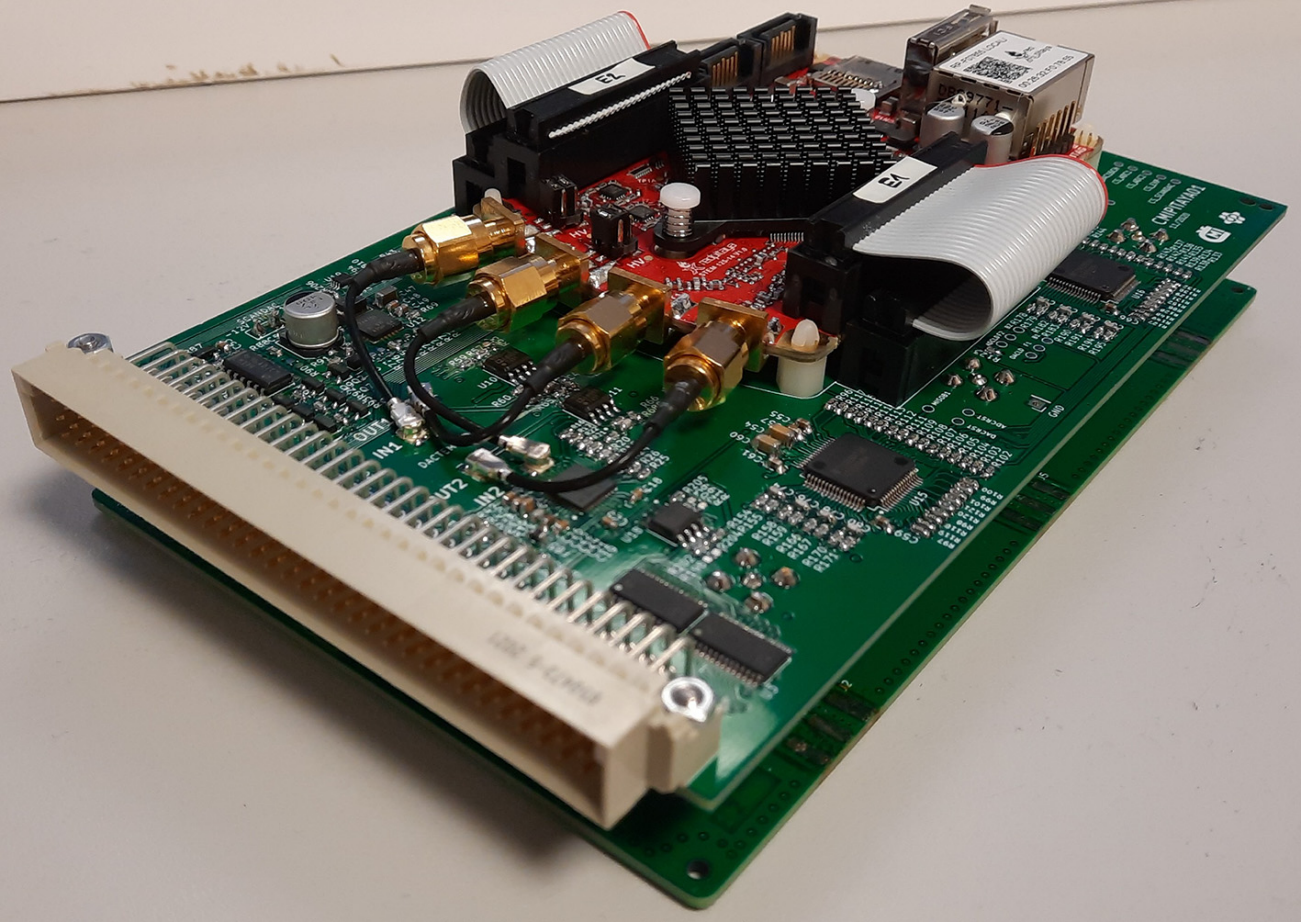
Photograph of the DSP - Red Pitaya and the signal acquisition and generation framework.

The DSP consists internally of two independent parts described below: a feedback loop module that is implemented in the Red Pitaya (RP) single board computer and a signal acquisition and generation framework that is formed by two electronics boards connected to RP via Serial Peripheral Interface (SPI) and includes one 16-channel 16-bit DAC, three single channel 20-bit DACs and two 8-channel 18-bit ADCs. RS232 interface is also included. Block schema of the DSP and an example showing how it could be used in a SPM system are shown in Fig. 1, Fig. 2, respectively. A photograph of the completed DSP is provided in Fig. 3. All the functionality is controlled by a single C language Application Programming Interface (API). For running a Scanning Probe Microscope, a server is provided for a client–server operation, created using this API, which allows the electronics to be controlled from a client outside of the RP (the graphical user interface to the microscope), connected to it via Ethernet.

### 2.1 Feedback loop module

The feedback loop is implemented on a Field Programmable Gate Array (FPGA) that is part of the Zynq chip on the Red Pitaya board. Instead of the FPGA resources provided with Red Pitaya it uses a custom built bitstream, created using Verilog language and Vivado Suite tools from Xilinx.

The main source of data for feedback loop is a 2-channel 14-bit AD converter (LTC2145-14) available on the Red Pitaya board, running at 125 MS/s. The sampled data can be used directly (e.g. in contact mode SPM operation) or passed through two independent lock-in amplifiers to obtain eventually amplitude (e.g. in tapping mode SPM operation) or phase (e.g. for frequency modulated SPM operation, together with a phase locked loop). Different digital feedback loops are implemented. The main feedback loop’s purpose is to serve as a source of *z* feedback for SPM measurements. Other feedback loops can be used to eventually provide phase-locked loop feedback for frequency modulated SPM and to maintain the oscillations amplitude constant in tapping mode or frequency modulated SPM. Some other slower feedback loops (e.g. for Kelvin Probe Force Microscopy) are available in the server, running on the processor side. Output of the feedback loop, designed for driving the scanner in the *z* direction, can be passed to the microscope using the 2-channel 14-bit DA converter (AD9767) available on the Red Pitaya board, running at 125 MS/s, or using a 20-bit DA converter that is connected directly to the FPGA using SPI interface.

### 2.2 Signal acquisition and generation framework

We have designed two printed circuit boards to enhance Red Pitaya’s capabilities. The main board, denoted as CMIPITAYA, has an Eurocard form factor (160 mm × 100 mm) with 96pin Euro connector. It needs to be supplied by  ± 12 V for the analog part and  + 5 V for Red Pitaya. It was designed for  ± 10 V input and output voltages. A Red Pitaya is mounted on top of this board and is connected by two flat cables using its extension connectors. The board features two 8-channel, simultaneously sampling 18-bit AD converters from Texas Instruments (2 × ADS8598H) and one 16-channel 16-bit DA converter also from Texas Instruments (DAC81416). Input signals for Red Pitaya are routed by using two multiplexers (MUX36S16), one for each input. Communication between Red Pitaya and the converters uses SPI interface driven from the Linux OS on Red Pitaya. There is also RS232 interface that can be used for controlling piezo amplifiers with digital inputs. The I2C interface is also present, however it is not used now.

The other board (CMIDAC11001) has same form factor as the main board and can be used separately or in combination with the main board. It features three DA converters from Texas Instruments namely the DAC11001, which are single channel DAC with 20-bit resolution, 1-LSB DNL and 4-LSB INL. We chose LTC6655LN from Analog Devices as references, one for each channel. The board requires  ± 12 V and 3.3 V power supplies. Output voltages are jumper configurable to  ± 10 V, 0–10 V, ±5 V and 0–5 V, respectively. In current setup we use SPI communication which is implemented directly in the FPGA of Red Pitaya. This ensures much faster and non-interrupted transfers compared to CPU SPI link. All three DACs are connected in a daisy-chain, hence all voltages can be set within a single SPI message. Nevertheless, we have provided a separated connector for communication with only one of the converters, if needed. All the voltages are accessible via SMA connectors or via the Euro96 connector if used in combination with the main board.

The three 20-bit DACs were designed to be used to control the scanning process, i.e. the x,y and *z* positions of the scanner, to be applied on the piezoelectric scanner elements, after appropriate amplification. Nevertheless, they can be used as a general purpose high resolution DAC. Voltage in each channel can be set independently via API.

### 2.3 Server

All the functionality of the feedback loop module and signal acquisition framework are controlled using a dedicated library written in C, which provides API for all the necessary operations: setting parameters for the feedback loop on FPGA, controlling the lock-in amplifiers, collecting the analog data from AD converters and driving DA converters or serial communication for microscope scanner. Using this library, a server is created, whose purpose is to provide communication interface for microscope control software set up on user’s side, which can be of different complexity, ranging from a small script to a complex graphical user interface with live view on all the scanning parameters and data. The server operation is driven by timer interrupts that are periodically updating the values read from AD converters or FPGA and passing the data to DA converters. The maximum possible frequency of timer interrupts is the main bottleneck of using the server, run on a processor, for the SPM control. Depending on the load on the SPI interface, which is the key limiting factor, the speed of this periodic update loop is 2–10 kHz. It should be however noted that this limitation does not apply to the feedback loop which keeps the probe-sample position constant, which is performed independently on the FPGA with a few orders of magnitude higher frequency.

The communication interface is realised over Ethernet using TCP sockets. Communication is done on a client–server basis, where the client (SPM control programme operated by user) sends a message to the server and gets a response. Messages are passed as GwyFileObject structures serialised by Gwyfile library [10], with a variable number of items inside, depending on the message type and user’s wishes on the number of parameters that should be passed at the moment. For deserialization, the Gwyfile library can be used again, or custom implementation based on the Gwyddion file format description can be done. Messages can be simple, e.g. to set the system into the feedback, or complex, e.g. to set all the feedback parameters or collect multiple arrays of scanned data.

For performing non-raster and adaptive scanning, the server has the following features:

• All the scanned data that are passed from server to client include the *x* and *y* coordinates, as well as the timestamp, which simplifies the data processing, including triangulation or drift detection.
• Scan paths precomputed using library like Gwyscan can be loaded on the server and scanned.
• Lua scripting interface can be used for creating adaptive scan paths within the server.
• Advanced statistical functions within the Lua scripting interface can be used to control the real time scan path decisions on basis of already scanned data.

### 2.4 Summary of features and benefits

The key features of the design described above are:

• Low cost DSP with high bit depth AD and DA converters.
• Capability of performing all the typical SPM operations, using optical lever or self-sensing probes and interfacing to different types of piezo amplifiers.
• Data compatible to general XYZ data processing framework in Gwyddion open source software.
• Lua scripting interface including support for local statistical analysis.

## 3 Design files summary

The design files are available from online repository – see the Specifications table. As it includes many files, here we summarize only the main groups of design files available:

• PCB design files in KiCad format,
• C sources for the server, including the library providing API to all the functionality,
• C sources for the client,
• documentation files (API reference),
• Verilog sources for the FPGA part of the software, including the files for Xilinx Vivado suite.

## 4 Bill of materials summary

The Bill of Materials is very long and is part of the electronics boards design files available at online repository. Here we mention only critical components:

• Red Pitaya STEMLab 125–14 board (382 €)
• DAC81416RHAT (58 €)
• 2× ADS8598HIPM (37 € each)
• 3× DAC11001APFBT (62 € each)
• 3× LTC6655LNBHMS8-5 (66 € each)

## 5 Build instructions

To set up the DSP, the following steps should be performed:

1. Decide if you will use the CMIDAC11001 board separately or in connection with CMIPITAYA board and manufacture the printed circuit boards accordingly.
2. Connect the Red Pitaya and optionally CMIDAC11001 boards to the CMIPITAYA board.
3. Power up the system. If all voltages are within their tolerances, POWER_OK leds will light up.
4. Connect the Red Pitaya board to LAN and log into the OS. Install prerequisities needed by the server on the Red Pitaya board. This includes Lua5.3 and FFTW3 development libraries.
5. Download the gwyhwserver folder to the Red Pitaya board and compile it using make.
6. Test the functionality of all the connections by running provided examples. For example SPI communication with ADC and DAC can be tested with test_spi.c example.
7. Optionally, perform calibration of Red Pitaya’s inputs and outputs. See cal_inputs.c and cal_outputs.c for details.

## 6 Operation instructions

To use the presented DSP for scanning a sample surface, you need to have an additional hardware to make the system a complete Scanning Probe Microscope. This can be of different complexity and cost and it means having some variant of the following devices:

• Sensing element for probe-sample interaction. It can be a conventional optical lever based SPM cantilever probe (available from many providers) together with optical pickup components to detect how much the probe bends due to probe-sample forces (using laser, focusing optics and position sensitive detector). Alternatively, it can be a self-sensing probe with suitable pre-amplifier, e.g. Akiyama probe with pre-amplifier board from Nanosensors company. DC voltage or AC signal from the sensing element should be connected to one of the DSP inputs.
• Scanner capable of performing motion with high spatial resolution in all the three directions, x,y and *z*. It can be a dedicated positioning system (made e.g. by Physik Instrumente company as in our case) or just a set of piezoelectric transducers mounted in the three directions. Usually the *x* and *y* directions are used for scanning while the *z* direction corresponds to surface normal and the feedback to keep the probe-sample interaction constant is established in this direction.
• Scanner driving system, either a digital piezo controller or a set of high voltage amplifiers with analog inputs. The x,y and *z* outputs of the DSP should be connected to it. Additional signals might be connected as well, depending on the scanning regime, e.g. an AC signal for mechanically driving the cantilever vibrations in tapping mode operation.

You also need a client software to interact with the DSP. The client software uses communication interface commands described in the documentation available at the online repository to control the experiment. This includes e.g. providing the path that should be scanned, letting the system to start the feedback loop and adjusting its parameters, adjusting scanning speed and reading the scanned data. An example of such a client software is provided as well (Gwyscope).

The key steps to run the presented DSP with the parts listed above are as follows:

1. Change directory to the gwyhwserver folder and set up the hwserver.ini file to reflect the actual hardware: range of the amplifiers, type of the sensing element, input multiplexers, calibration data for Red Pitaya board, etc. We provide a sample of our .ini file for reference.
2. Start server using command

**Table:** 

./hwserver [port] [ini_file]
for example
./hwserver 50100 hwserver.ini
3. Connect to the server using the client and perform the SPM operations.

## 7 Validation and characterization

### 7.1 General performance

Performance of a SPM DSP itself is only one aspect of the complete process of performing a good SPM measurement, where all the possible sources of electrical and mechanical noise play a role. Therefore we demonstrate the capability of performing real measurements with two hardware setups. The first presented setup is based on a conventional, optical pickup based, custom built AFM head, with tapping mode probe (RTESPA-300 by Bruker), a 2D positioning system using two P-622K110 actuators (Physik Instrumente) and a digital closed loop piezo controller E-727 (Physik Instrumente). The second setup uses Piezo-Resistive Sensing & Active (PRSA) self-sensing probes (PRSA-L100-F400-Si-PCB by SCL-Sensor.Tech) in a commercial Nenovision SPM hardware LiteScope. The LiteScope scanning head is designed to be used together with Scanning Electron Microscopes for correlative measurement, and its electronics was created by Nenovision to use the presented DSP.

The two systems were tested by comparison to a state of the art commercial SPM (Dimension Icon, Bruker) using tapping mode and RTESPA-525 probes. To show the performance at ultimate resolution we used silicon steps sample, manufactured at Physikalisch Technische Bundestanstalt, Germany. Mono atomic silicon steps are one possible realisation of metre, as defined in 2019 *Mise en Pratique* [11], where they are suggested for the calibration of the vertical axis of microscopes such as SPMs [12]. According to Committee on Data for Science and Technology CODATA [13], the height of atomic steps can be calculated as $d_{111}=313.560\;115\;1\times 10^{-12}\;m$, with a standard uncertainty of $0.000\;005\;3\times 10^{-12}\;m$, which provides far the best achievable artefact for SPM calibration for very small *z* ranges. We used the silicon steps to calibrate the presented setups and then evaluated the noise levels and overall performance of the microscopes.

**Fig. 4:**
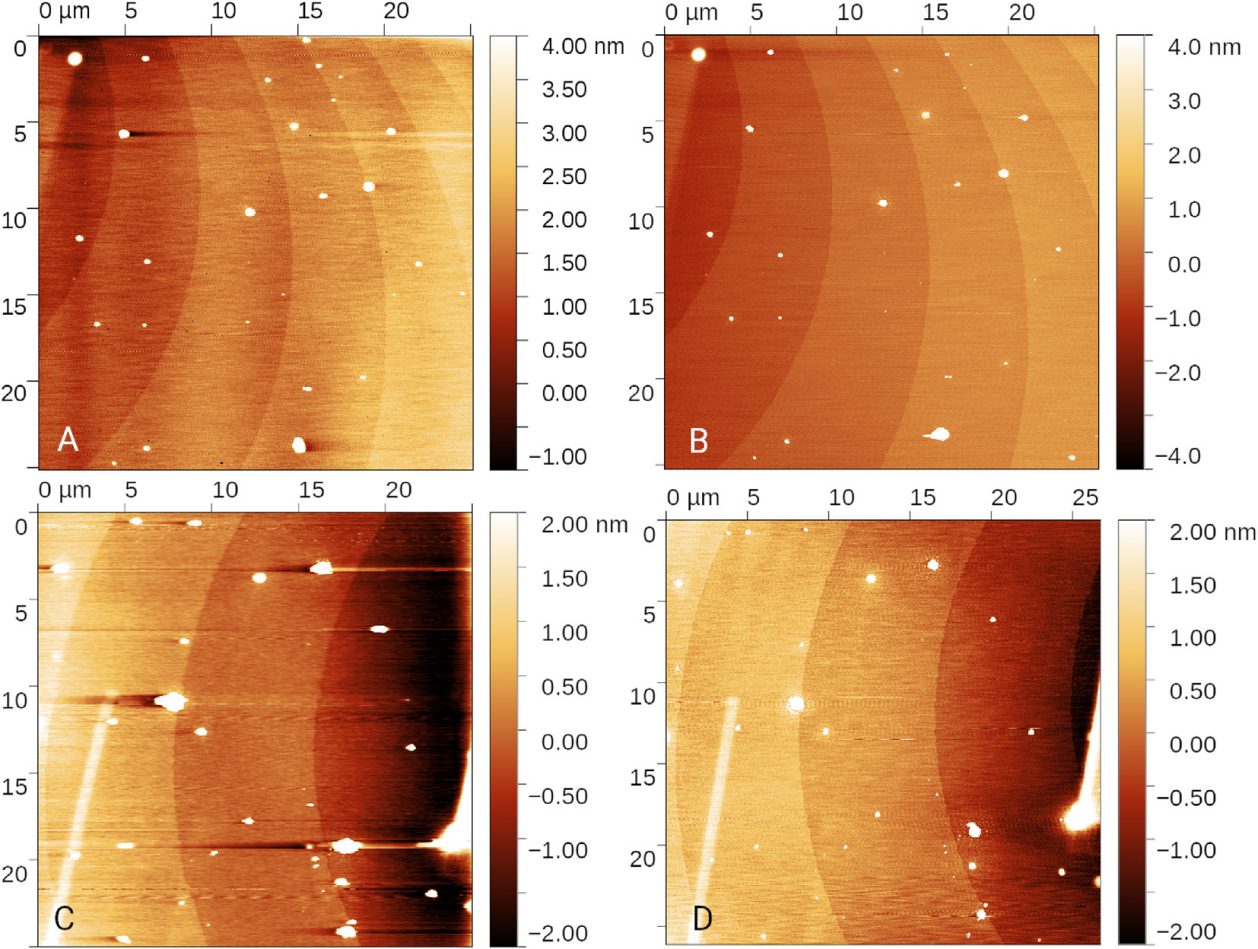
Silicon steps result, measured on custom built setup with non-contact probe and deflection based setup (A), NenoVision LiteScope with PRSA self-sensing probe (C) and the same areas measured on Dimension Icon (B, D) in the tapping mode.

In Fig. 4 the results obtained by both systems for atomic steps are shown and compared to the commercial instrument. Silicon steps can be clearly seen even though the sample, which is approximately 20 years old, is already covered by some dust particles. Edges of rectangular areas of contamination from inspection on an electron microscope can be also seen and were used, together with the dust particles, as a guidance to find the same scan areas. The sample was oriented to make the fast scan axis approximately perpendicular to the step edges in order to minimize the impact of temperature drifts during the measurement. Line by line leveling was used to remove the residual drifts. All three microscopes were kept in an enclosure to reduce both acoustic noise and temperature fluctuations from the air conditioning. Both data were processed in the same manner (1D profiles leveling after masking out the dust particles, 2D polynomial background subtraction to remove the scanner bow, followed by three point leveling to get the mean plane). The noise level in data from Czech Metrology Institute’s custom built system is 225 pm; in the mechanically better optimised LiteScope system it is below 100 pm. So the DSP can achieve noise levels comparable to state of the art microscopes. Nevertheless, noises at this level can be dominated by probe, environment, scan parameters, scan speed and even the user experience, so their absolute values are not to be compared. Note that in the case of the presented DSP we did not need to decrease the scanner range (as set to 1 μm on Dimension Icon), as the 20-bit DA converter running the *z* piezo amplifier has sufficient bit depth for the full scanner range (10 μm in custom build set-up, or 20 μm in LiteScope). Although further optimisations of the scan parameters could still be done, the data obtained by all the systems based on the presented DSP are already good enough for the silicon steps height evaluation based on background separation used in Ref. [14], which is the intended use of this type of sample.

### 7.2 Adaptive scanning using Lua scripting language

To demonstrate the capability of creating adaptive scripts based on local statistical information we present two examples. The first script chooses adaptively the distance between subsequent profiles in a roughness measurement. When measuring surface roughness, we are interested in height and lateral scale information, which can both be evaluated using the Power Spectral Density Function (PSDF), computed from the profiles via Fast Fourier Transform. However, profiles closer than the correlation length are highly correlated and contribute largely redundant data [15], [16], [17]. The script aims to collect the maximum of independent statistical information at minimal time by avoiding the acquisition of redundant profiles. To determine how far the next measured profile should be, it uses the correlation length estimated at the controller level using a function get_correlation_length() accessible from Lua.

**Fig. 5:**
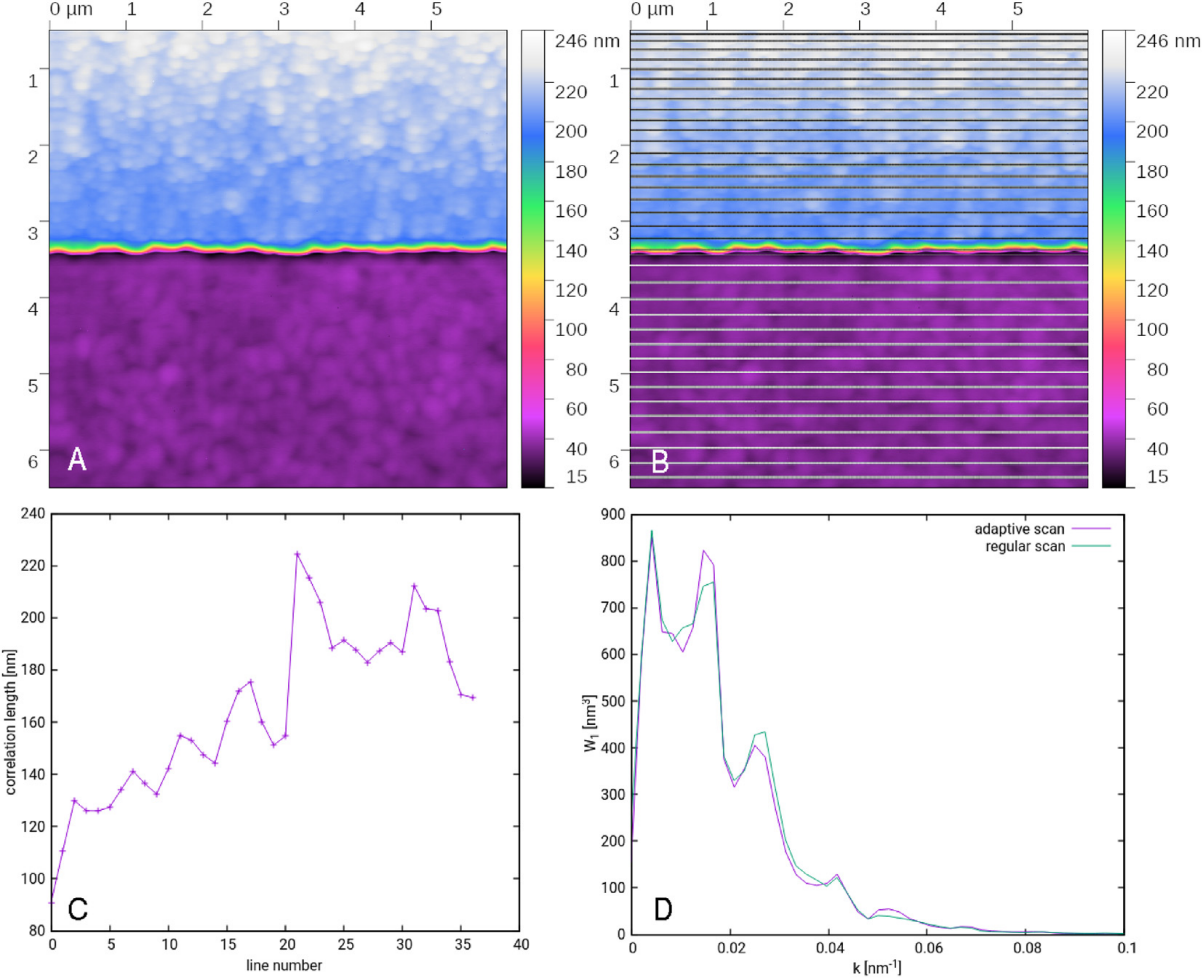
Sample with two different roughnesses: A) regular scan, B) illustration where the adaptive scan decided to take profiles, C) estimated correlation lengths, D) comparison of the power spectral density at upper half of the sample obtained from regular and from adaptive scan.

A measurement of an edge on a custom built grating is shown in Fig. 5. The grating is highly non-ideal, showing large surface roughness on both sides of the edge, with different statistical properties at the top and at the bottom part. This can be used to demonstrate the correlation length driven adaptive scanning. The profile positions measured in the adaptive scan are shown in Fig. 5B and the correlation length that was used to determine them can be seen in Fig. 5C. From Fig. 5D we can see that the resulting PSDF remains nearly same even though we sped up the measurement by factor of 30.

**Table:** 

-- Parameters
xrange, yrange = 6e-6, 6e-6
xoff, yoff = 0.0, 0.0
dx = 0.012e-6
-- Scan
gws_clear(p)
y = 0.0
Tavg = −1.0
xres = xrange/dx + 1
n = 0
repeat
gws_move_to(p, xoff, yoff + y, 0)
n = gws_scan_and_store(p, xoff + xrange, yoff + y, xres, 0)
Tpix = gws_get_correlation_length(p, n - xres, n)
Tavg = (Tavg > 0.0) and 0.75*Tavg + 0.25*Tpix or Tpix
y = y + Tavg*dx -- Move about one correlation length down
until y >= yrange

The second example is related to measurement of a diffraction grating. Grating pitch is frequently evaluated using SPM and there are many papers dealing with the methodology. One of the ways how to reduce the uncertainty is to measure in the fast scan axis only, making the grating perpendicular to it. Here we show a Lua script performing simple self-alignment of the fast scan axis during measurement. The script first scans several test profiles at different angles and after determining the grating orientation it performs a full image scan. For this a helper Lua function gws_get_optimum_angle() for fitting the minimum of period from several scan profiles at different angles was created.

**Table:** 

local function scan_line_at_angle(p, xcentre, ycentre, range, angle, res)
ca, sa = math.cos(angle), math.sin(angle)
xstart = xcentre + range/2 - range/2*ca
ystart = ycentre - range/2*sa
xend = xcentre + range/2 + range/2*ca
yend = ycentre + range/2*sa
gws_move_to(p, xstart, ystart, 0)
return gws_scan_and_store(p, xend, yend, res, 0)
end
-- Parameters
range = 20e-6
res = 500
xoff, yoff = 1e-6, 1e-6
-- Scan angles for alignment
y = yoff + range/2
dx = range/(res - 1)
angle_scan = {}
n = −1
gws_clear(p)
for angle = −0.1, 0.1, 0.05 do
angle_scan[#angle_scan + 1] = {start = n + 1, npts = res, angle = angle}
n = gws_scan_at_angle_and_store(p, xoff, yoff + y, range, angle, res)
end
angle = gws_get_optimum_angle(p, angle_scan)
-- Scan image with the found angle
for y = 0, range, dx do
gws_scan_at_angle_and_store(p, xoff, yoff + y, range, angle, res)
end

**Fig. 6:**
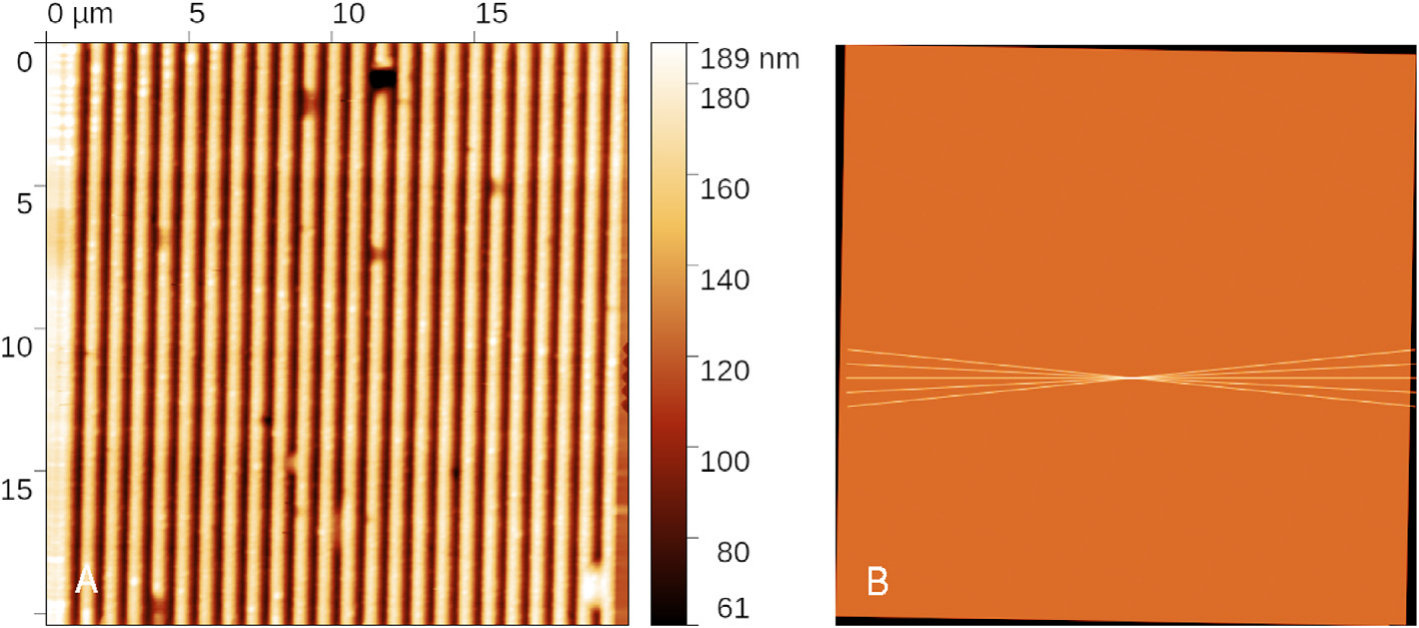
Diffraction grating measurement taken along the optimum direction (left) and measured data density showing both the initial set of five profiles that were used to determine the scan angle and a rotated square which was then used to measure the grating (right).

Measurement results can be seen in Fig. 6. All the scanned profiles are perpendicular to the grating edges, which means that the 1D algorithms for grating evaluation can be used. The result is shown in absolute coordinates, i.e. it still appears rotated. Note, however, how the orientation of the final scanned rectangle in Fig. 6B matches the grating orientation in Fig. 6A.

The two examples were relatively simple for the purpose of illustration. Nevertheless, since the controller is Turing-complete, it is possible to envision more complex scripts. A Lua implementation of Hilbert curve scanning paths, replicating those generated by Gwyscan, is included in the source code as an example of scanning not based on straight profiles. Using a local information density estimation, a Lua controller script could even automatically perform an adaptive 2D scan, focusing on areas with high information density. The processing power is also sufficient for estimation of quantities using an already trained neural network, where the training would of course be done on the workstation.

### 7.3 Performance summary

To summarize, the main capabilities of the presented DSP are as follows:

• Form factor: Eurocard (160 mm × 100 mm), three slots, 96 pin Euro connector
• Power supply: 5 V (2 A), ±12 V (1 A)
• Input: DC coupling, 16-channel, 18-bit, simultaneous sampling ADC (2× ADS8598H), range ±10 V or ±1 V (software configurable) and fast DC coupled 2-channel 14-bit ADC (LTC2145-14) available on the Red Pitaya board
• Output: DC coupling, 16-channel, 16-bit DAC (DAC81416), range 0–5 V, 0–10 V, ±2.5 V, ±5 V, ±10 V (software configurable), additional 3-channel, 20-bit DAC (3× DAC11001A), range 0–5 V, 0–10 V, ±5 V, ±10 V (jumper configurable) and fast DC coupled 2-channel 14-bit DAC (AD9767) available on the Red Pitaya board
• 2 independent lock-in units, 3 signal generators, 5 PID controllers
• Ethernet (1 Gbit/s), USB 2.0 (480 Mbit/s)
• Probe types: optical pickup based, piezoresistive (pre-amplifier needed), self-sensing, Scanning Tunneling Microscopy (pre-amplifier needed).
• Supported scanner types: analog (0–5 V, 0–10 V, ±5 V, ±10 V configurable), digital using RS232 serial interface, PI General Command Set, general GwyFileObject message based digital interface for custom built controllers.
• Feedback regimes: DC proportional to error signal, AC tapping amplitude and phase, phase locked loop (frequency modulated SPM).
• Support for non-raster scanning as described in the previous sections of this paper.

## CRediT authorship contribution statement

**Miroslav Valtr:** Conceptualization, Methodology, Software, Writing - review & editing. **Petr Klapetek:** Conceptualization, Methodology, Software, Validation, Writing - original draft. **Jan Martinek:** Methodology, Investigation. **Ond**ř**ej Novotn**ý**:** Validation. **Zden**ě**k Jel**í**nek:** Validation. **Václav Hortv**í**k:** Resources. **David Nečas:** Methodology, Investigation, Software, Writing - review & editing.

## Declaration of Competing Interest

The authors declare that they have no known competing financial interests or personal relationships that could have appeared to influence the work reported in this paper.
